# Supplementary material for: The adaptive stochasticity hypothesis: Modeling equifinality, multifinality, and adaptation to adversity
Source: Proc Natl Acad Sci U S A. 2023 Oct 10;120(42):e2307508120. doi: 10.1073/pnas.2307508120 (PMC10589678; doi:10.1073/pnas.2307508120)
Supplement: Supplementary file 1 — Appendix 01 (PDF) [file pnas.2307508120.sapp.pdf]

SUPPLEMENTARY MATERIAL

The adaptive stochasticity hypothesis: modelling equifinality, multifinality, and adaptation to adversity

Sofia Carozza[1,2]*, Danyal Akarca[1]*, Duncan Astle[1,3]
*Co-lead authors

1. MRC Cognition and Brain Sciences Unit, University of Cambridge, Cambridge, UK
2. Harvard Medical School, Cambridge, USA
3. Department of Psychiatry, University of Cambridge, Cambridge, UK

Corresponding authors:
Sofia Carozza (sofia.carozza@mrc-cbu.cam.ac.uk)
Danyal Akarca (danyal.akarca@mrc-cbu.cam.ac.uk)

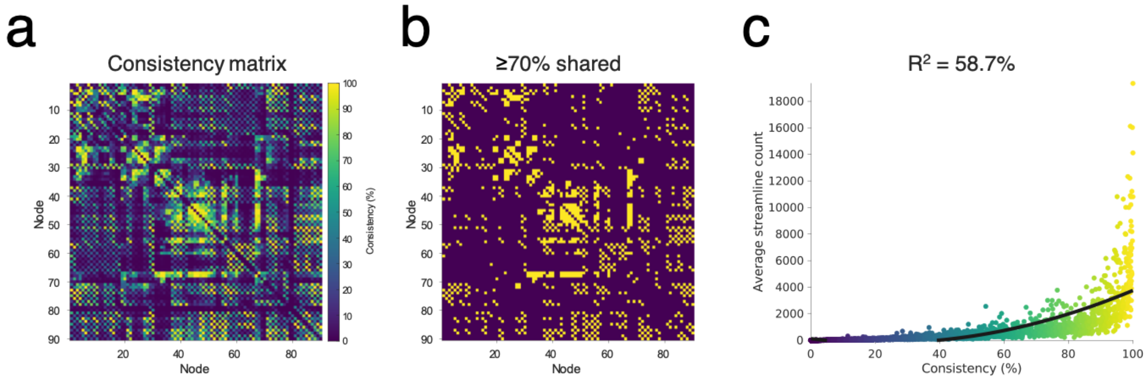

Supplementary Figure 1. Thresholding *dHCP* neonatal connectomes through consistency-based thresholding of the *n* = 685 *dHCP* neonatal connectomes. (a) The consistency matrix of how common an edge is across the sample. (b) The connectivity matrix, filtered to include only those edges which exist in ≥ 70% (*n* ≥ 480 of the sample). (c) The consistency of the edge across the sample is positively related to the average streamline count of that edge. A quadratic model fit to the data gives R^2 = 58.7%.

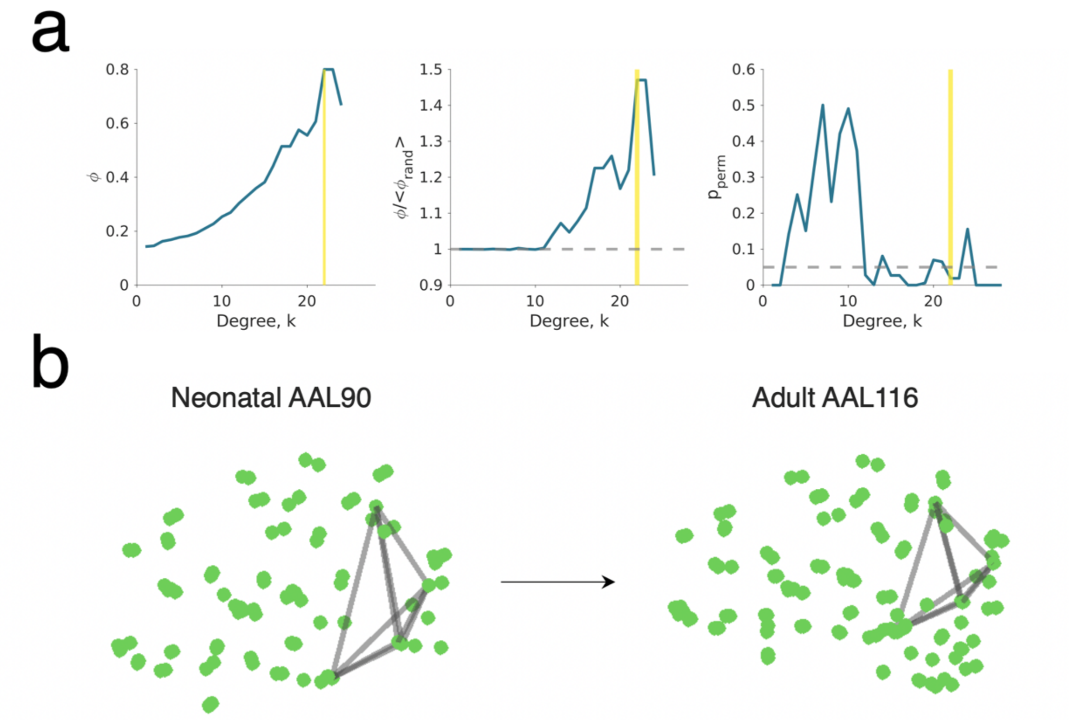

Supplementary Figure 2. Neonatal rich club topology as the generative model seed. (a) The rich-club coefficient chart for increasing degree, k thresholds. The selected threshold of k = 22 is highlighted, showing the highest ϕ = 0.8 (left). The normalized rich-club coefficient chart for increasing degree, k thresholds. Normalization was performed using the mean of 1000 randomized degree-preserving null models. The selected threshold of k = 22 is highlighted showing the highest $\phi/<\phi_{rand}>$ = 1.5 (middle). The permuted p-value (p_{perm}) chart for increasing degree, k thresholds. The selected threshold of k = 22 is highlighted showing significance; p_{perm} = 0.016 (right). (b) A graphical representation of the neonatal rich club in AAL90 MNI space, used as the seed network for generative modelling.

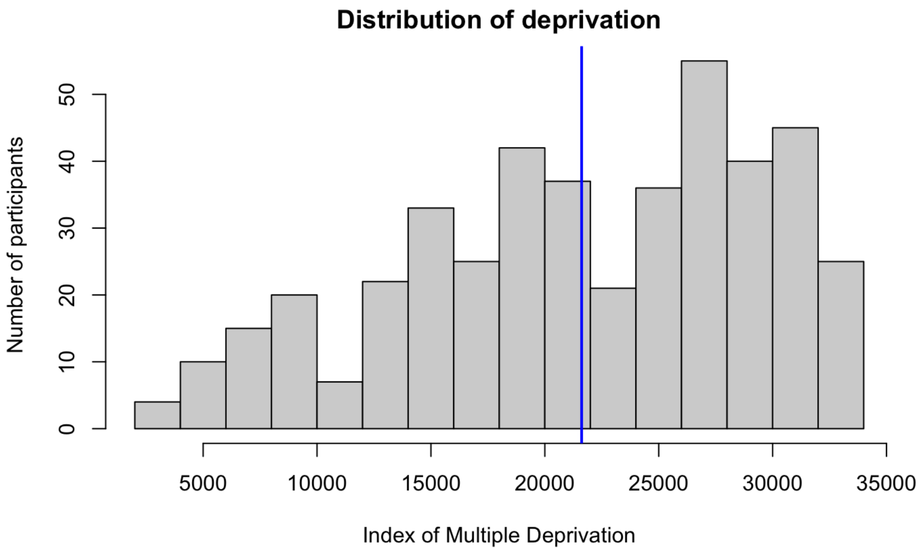

Supplementary Figure 3. Distribution of values of the index of multiple deprivation (IMD), a measure of the relative deprivation of the participant's area. The IMD accounts for income, employment, education, health, crime, barriers to housing and services, and the quality of the living environment. The blue line indicates the mean of the sample.

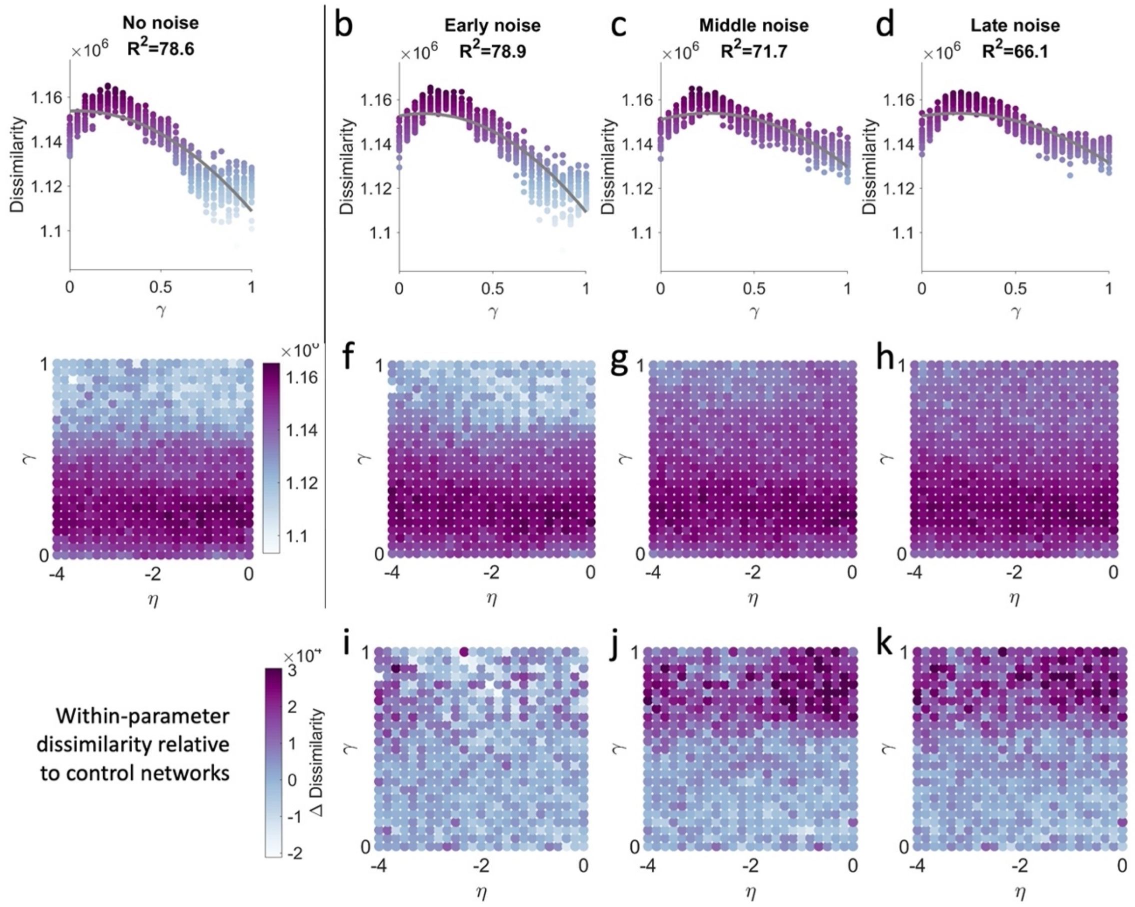

Supplementary Figure 4. Stochasticity later in network development results in greater variability in network topology. Compared to simulations run without noise (a) simulations with noise injected exhibit greater topological dissimilarity. Compared to (b) early noise, (c) middle and (d) late noise have a greater impact. When considering a heatmap of dissimilarity across the parameter space, the greatest increase in dissimilarity is localized at higher values of γ (e-k).

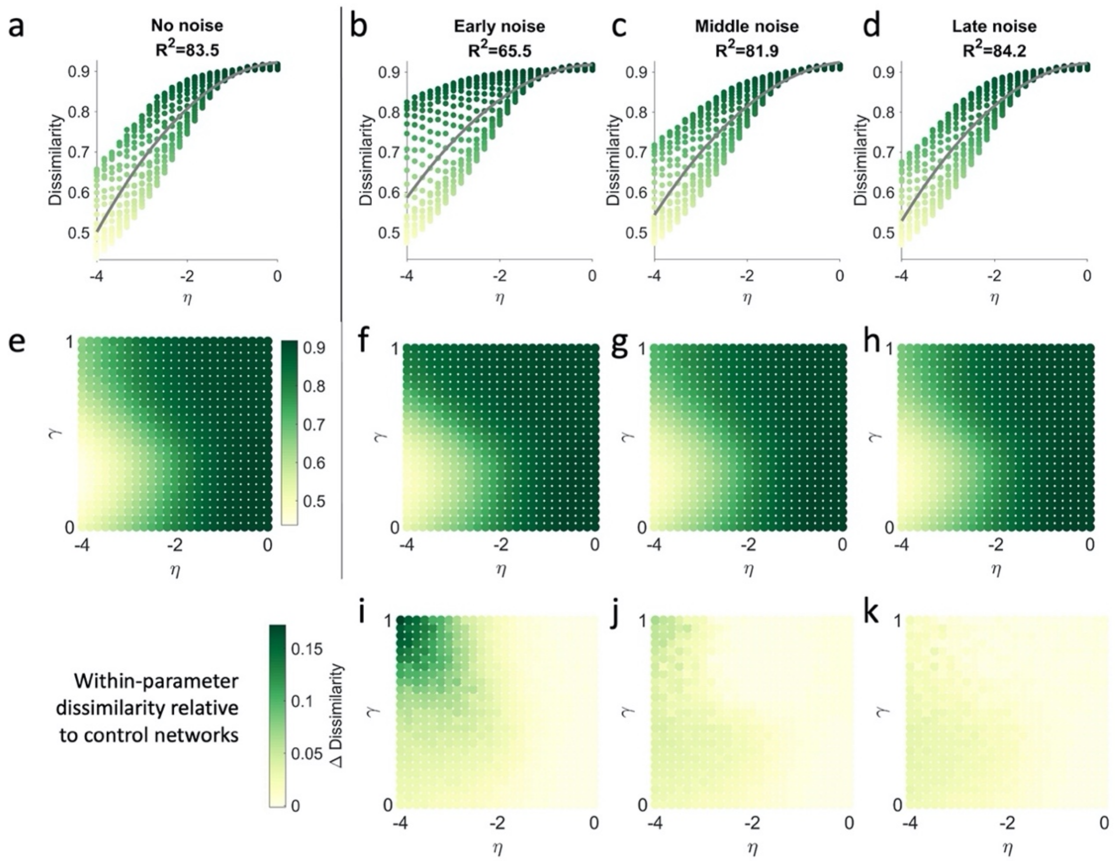

Supplementary Figure 5. Stochasticity later in network development results in greater variability in the consistency of connection. Compared to simulations run without noise (a) simulations with noise injected exhibit greater dissimilarity in embedding. Compared to (b) early noise, (c) middle and (d) late noise have a lesser impact. When considering a heatmap of dissimilarity across the parameter space, the greatest increase in dissimilarity is localized at lower values of η (e-k).

Random attacks

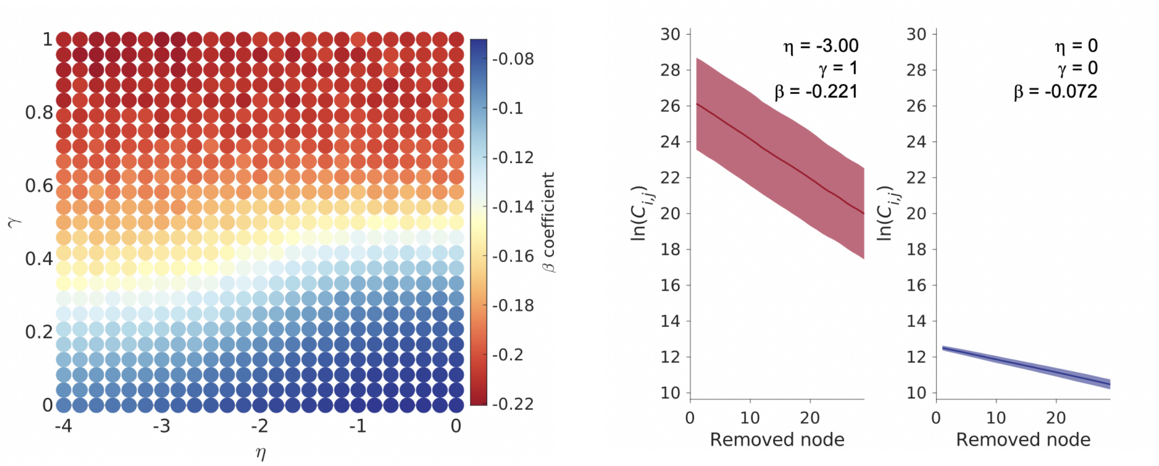

Supplementary Figure 6. Robustness trade-offs in network connectivity following random attacks. The β coefficient computed from a random attack regime is provided, showing that higher parameter (top left of the left landscape) networks have less robustness as the gradient of change is greater and vice versa (bottom right of the left landscape). Example network regimes are highlighted to the right, showing outcomes with the least (left) and most (right) robustness to change.

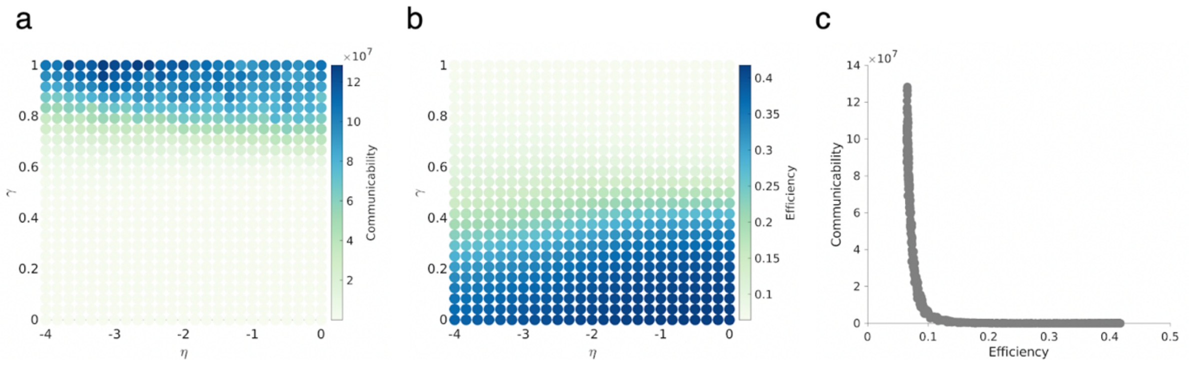

Supplementary Figure 7. The trade-off between network communicability and efficiency. (a) Network communicability is given across the wiring parameter space. Dark blue corresponds to high communicability while light green corresponds to low communicability networks. (b) The network efficiency is given across the wiring parameter space. Dark blue corresponds to high-efficiency networks while light green corresponds to low-efficiency networks. (c) The relationship between communicability and efficiency is given, showing a clear trade-off between the two.

	Pre-Imputation (*n* = 437)	Post-Imputation (*n* = 437)
	**Age (months)**	
min	62	62
max	223	223
mean(sd)	118.73 ± 27.81	118.73 ± 27.81
	**Sex**	
Male	285 (65)	285 (65)
Female	152 (35)	152 (35)
	**Index of Multiple Deprivation**	
min	2208	2208
max	32840	32840
mean(sd)	21,635.58 ± 7,795.45	21,615.05 ± 7,711.62

Supplementary Table 1. Descriptive statistics of the demographics of the CALM cohort.
